# Effects of decompressive cervical surgery on blood pressure in cervical spondylosis patients with hypertension: a time series cohort study

**DOI:** 10.1186/s12893-015-0117-y

**Published:** 2016-01-06

**Authors:** Hong Liu, Hai-Bo Wang, Lin Wu, Shi-Jun Wang, Ze-Chuan Yang, Run-Yi Ma, Kathleen H. Reilly, Xiao-Yan Yan, Ping Ji, Yang-feng Wu

**Affiliations:** Department of Orthopaedic Surgery, Peking University First Hospital, Xishikuda Street 8#, Xicheng Dist, Beijing, 100034 P.R. China; Peking University Clinical Research Institute, Xueyuan Rd 38#, Haidian Dist, Beijing, 100191 P.R. China; Department of Cardiology, Peking University First Hospital, Xishenkuda Street 8#, Xicheng Dist, Beijing, 100034 P.R. China; Department of Epidemiology and Biostatistics, School of Public Health, Peking University Health Science Center, Xueyuan Rd 38#, Haidian Dist, Beijing, 100191 P.R. China; Independent Consultant, New York City, NY USA; The George Institute for Global Health at Peking University Health Science Center, No. 6 Zhichun Road, Beijing, 100083 P.R. China

**Keywords:** Cervical spondylosis myelopathy, Cervical decompressive surgery, Hypertension, 24 h ambulatory blood pressure measurement

## Abstract

**Background:**

Patients with cervical spondylosis myelopathy (CSM) and complicated with hypertension are often experiencing a blood pressure decrease after taking cervical decompressive surgery in clinical observations, but how this blood pressure reduction is associated with the surgery, which cut cervical sympathetic nervous, has never been rigorously assessed. Thus, the purpose of this study is to investigate the effect of cervical decompressive surgery on blood pressure among CSM patients with hypertension.

**Methods/Design:**

The study will be a time series cohort study. Fifty eligible patients will be selected consecutively from the Peking University First Hospital. Two 24-h ambulatory blood pressure measurement (ABPM) will be taken before the surgery, apart by at least 3 days. The patients will be followed up for another two ABPMs at 1 and 3 months after the surgery.

We will recruit subjects with cervical spondylosis myelopathy meeting operation indications and scheduled for receiving cervical decompressive surgery, aged 18–84 years, have a history of hypertension or office systolic blood pressure ≥140 mmHg on initial screening, and willing to participate in the study and provide informed consent. Exclusion criteria includes a history of known secondary hypertension, visual analogue scale (VAS) score ≥4, and unable to comply with study due to severe psychosis.

The change in systolic ABPs over the four times will be analyzed to observe the overall pattern of the blood pressure change in relation to the surgery, but the primary analysis will be the comparison of systolic ABP between the 2^nd^ and 3^rd^ , 4^th^ measurements (before and after the surgery). We will also calculate the regression-to-the-mean adjusted changes in systolic ABP as sensitivity analysis. Secondary endpoints are the changes in 24 h ABPM diastolic blood pressure, blood pressure control status, the use and dose adjustment of antihypertensive medication, and the incidence of operative complications. Primary outcome analyses will be carried out using analysis of covariance, as well as the first secondary endpoint.

**Discussion:**

This study will inform us the important knowledge about cervical sympathetic nervous system (SNS) and blood pressure. Once confirmed, it may help to produce new method for control of hypertension, which is the leading cause of death in the world.

**Trial registration:**

The study is registered to Clinical Trials.gov (NCT02016768).

## Background

Hypertension is a major global health concern. It is estimated that 30–40 % of the adult population in the developed world are suffering from hypertension [1, 2]. Moreover, incidence and prevalence of hypertension are increasing, especially in developing countries. The effects of current pharmacologic treatment of hypertension remain suboptimal in both developing and developed countries [3, 4]. The common causes responsible for the poor control of high blood pressure are attributed to physicians’ attitudes and patient non-compliance to lifelong pharmacological therapy for asymptomatic hypertension [5–7]. Moreover, hypertension that remains uncontrolled in spite of the use of 3 or more antihypertensive drugs has been called resistant hypertension [8]. Clinical evidence indicates that the risk of major cardiovascular events is higher among patients with resistant hypertension than that among patients with controlled blood pressure [5]. Thus, developing novel approaches for management of hypertension, especially for resistant hypertension, is an important issue for the future of the treatment of hypertension.

It is well known that sympathetic nerve fibers are rich in cervical spinal tissues [9–11]. Yamada et al. [9] reported that there was different sympathetic innervation between the cervical dura mater and the posterior longitudinal ligament. It seems reasonable that the compression or irritation of cervical dura mater and/or posterior longitudinal ligament may cause an increase in blood pressure or aggravate pre-existing hypertension among patients with cervical spondylosis myelopathy.

Tamura [12] found that neck injury, such as whiplash, may lead to the cranial symptoms of the Barré-Lieou syndrome (including a group of symptoms such as headache, vertigo, tinnitus and ocular problems), which may result from irritation of the sympathetic nervous supply. We hypothesize that the cervical sympathetic nerve is also associated with the occurrence of resistant hypertension and that cervical decompressive surgery may be beneficial in blood pressure control among patients with cervical spondylosis myelopathy [13]. The purpose of this study is to investigate the effect of cervical decompressive surgery on resistant hypertension based on a novel mechanistic hypothesis of essential hypertension.

## Methods and design

### Study design and Setting

**Fig. 1:**
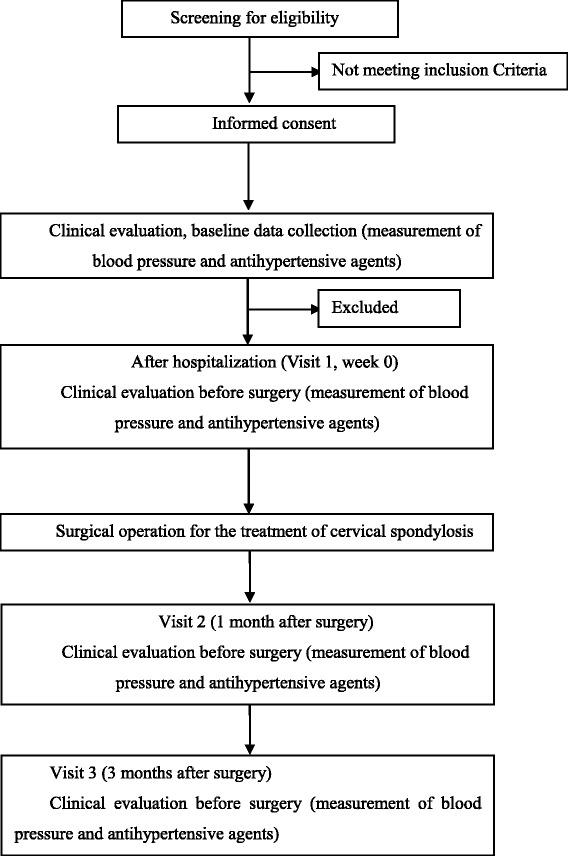
Study measures and time points

The study is a time series cohort study. Patients with both cervical spondylosis myelopathy and hypertension are invited to participate in the study and subsequently receive cervical decompressive surgery. Patients are recruited continuously by surgeons at the Department of Orthopaedics, Peking University First Hospital in which there are about 30–40 patients with both cervical spondylosis myelopathy and hypertension each year. These subjects will be followed up at 1 and 3 months post-operation after enrollment. The study procedures and informed consent form have been approved by the Institutional Review Boards of the Peking University Health Science Center in Beijing, China, and registered at NCT02016768. Figure 1 shows an overview of the most important procedures in the study.

### Participants

#### Inclusion criteria

The patients to be included in the study should meet the following inclusion criteria:

1. Patients with cervical spondylosis myelopathy, such as bilateral hand clumsiness and numbness, walking unsteady, weakness in the limbs, muscular dystrophy, increasing deep tendon reflex and positive Hoffmann and Babinski signs; the underlying diseases including multilevel cervical spinal stenosis, ossification of posterior longitudinal ligaments (OPLL), disc herniation, meeting operation indications: spinal neurological symptoms for at least 2 months, which are not cured by conservative treatment or in progression, significant spinal compression indicated by magnetic resonance imaging.
2. Aged 18–84 years.
3. Plans for receiving cervical decompressive surgery.
4. A history of hypertension or office systolic blood pressure ≥140 mmHg on initial screening.
5. Willing to participate in the study and sign informed consent.

#### Exclusion criteria

If one of the following criteria is met, patients will be excluded from the study:

1. A history of known secondary hypertension.
2. Visual analogue scale (VAS) score ≥4.
3. Unable to comply with study due to sever psychosis.

#### Recruitment and consent

The patients who suffer from cervical spondylosis myelopathy and plan for receiving cervical decompressive surgery will be selected for potential participation by the surgeons at the Department of Orthopedics. After consultation with inpatients and outpatients and screening their clinical information, the principal investigator of the study (the surgeon) informs patients whether they are eligible for the study. Informed consent is acquired immediately after screening for every subject by the principal investigator.

#### Withdrawal of individual subjects

Subjects can withdraw from the study at any time for any reason without any consequences. The investigator can also decide to release a subject from the study for medical reasons. For every subject who decides to withdraw from the study, the reasons for withdrawal will be recorded.

A total of 18 patients have given informed consent for participation and been enrolled into the study from July 2014 to Oct 2015.

### Cervical decompressive surgery

The cervical decompressive surgery will be done with one of the following four operating methods: international standard anterior cervical discectomy and fusion (standard Smith-Robinson technique, intervertebral disc incised and removed to reduce pressure, interbody fusion by implanting cervical interbody fusion cage), anterior cervical corpectomy and fusion (cervical corpectomy decompression, interbody fusion by implanting titanium cage), posterior bilateral open-door decompression (modified Hemo anchored method) or one-side open door decompression (modified Hirobayashi anchored method). The indications for anterior approach are subjects with less than three levels disc herniation, while for posterior approach are those with multilevel cervical spinal stenosis and continuous or mixed OPLL.

### Initial screening, assessment and follow-up

**Fig. 2:**
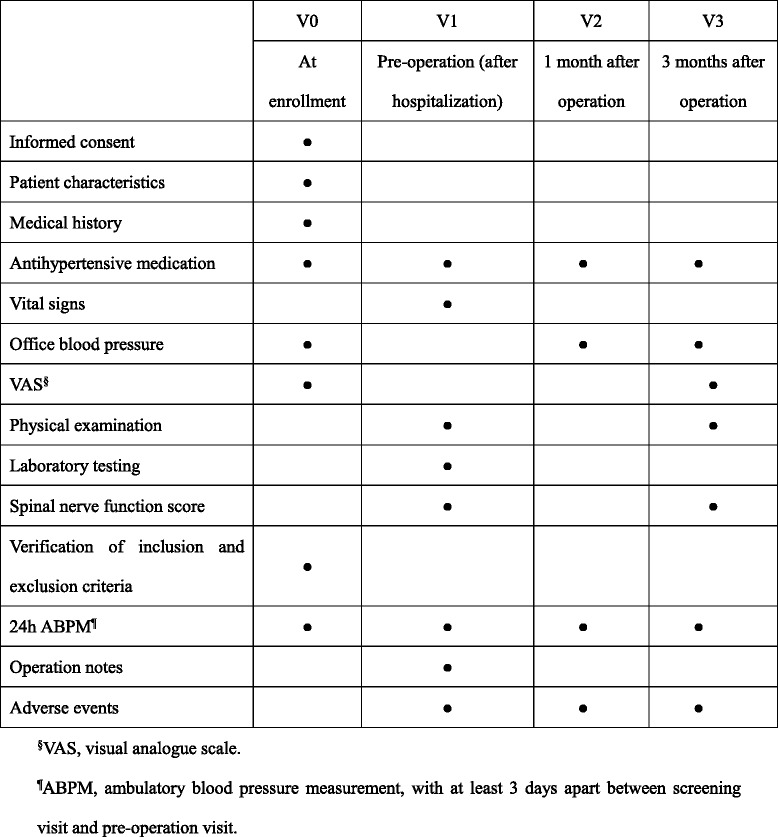
Observation, assessment, and follow-up schedule

After providing informed consent, potential participants will be asked standardized questions about their demographic characteristics and medical history (especially for the history of hypertension and antihypertensive therapy) at initial screening. In addition, office blood pressure, VAS score and 24 h ambulatory blood pressure measurement (ABPM) will also be measured and used for checking inclusion/exclusion criteria (Fig. 2).

Eligible participants will be hospitalized for surgical treatment, and a pre-operation visit and clinical evaluation will be done before surgery, including antihypertensive medication, vital signs, physical examination, laboratory testing, spinal nerve function score and 24 h ABPM (Fig. 2). It is required that there is at least 3 days apart between initial screening visit and pre-operation visit. As soon as the operation condition is met, these subjects will receive cervical decompressive surgery, and their operation notes and adverse events will be recorded in detail.

All subjects will be followed up at 1 and 3 months after surgical operation, with antihypertensive medication recording, measurement of office blood pressure, 24 h ABPM and adverse events assessment collected at each time. In addition, VAS, physical examination and spinal nerve function score will be measured and collected at 3 month follow-up.

Regular antihypertensive medication therapy will be continued during the entire study period, however, the investigator can adjust the antihypertensive medication if necessary, according to treatment needs of individual patients.

### Study endpoints

The primary endpoint is estimated as the difference between systolic ABPM before surgery (2^nd^ measurement) and the systolic ABPM at 1 month and 3 month post-operation (3^rd^ and 4^th^ measurement).

Four secondary endpoints were defined as: 1) The difference between diastolic ABPM before surgery and ABPM diastolic blood pressure at 1 month and 3 month post-operation; 2) control status of blood pressure at 1 month and 3 month post-operation (blood pressure control is achieved if systolic/diastolic blood pressure <140/90 mm Hg, reducing systolic blood pressure by >20 mm Hg or reducing diastolic blood pressure >5 mm Hg); 3) the use and dose adjustment of antihypertensive medication; and 4) the incidence of operative complications, including spinal nerve trauma, incision infection and fluid, pneumonia and urinary system infection etc.

### Sample size calculation

Based on previous study which explored the efficacy of renal sympathetic denervation in treating hypertension, we assume that for the current study the primary outcome (the change of 24 h ABPM systolic blood pressure from pre-operation to the measurement at 3 months post-operation) has a standard deviation of 15 mm Hg [14]. We wish to be able to detect a difference of 11 mm Hg. Assuming a 20 % drop-out rate, a total of 50 participants are required to provide 90 % power, with the risk of two-sided type І error of 0.01.

### Statistical analysis

Analyses will be made using SAS statistical software (version 9.3) by researchers at the Peking University Clinical Research Institute. The change in systolic ABPMs over the four times will be analyzed to observe the overall pattern of the blood pressure change in relation to the surgery, but the primary analysis will be the comparison of systolic ABPM between the 2^nd^ and 3^rd^, 4^th^ measurements (before and after the surgery). We will also calculate the regression-to-the-mean adjusted changes in systolic ABPMs as sensitivity analysis. Primary endpoint analyses and the change in systolic ABPMs over the four times will be carried out using analysis of covariance, as well as the first secondary endpoint (the change of 24 h ABPM diastolic blood pressure). Descriptive statistics will be used to summarize control status of blood pressure, the use and dose adjustment of antihypertensive medication and the incidence of operative complications.

## Discussion

The etiology and mechanism of essential hypertension remains unclear. Non-operative methods of essential hypertension treatment including antihypertensive pharmacologic drugs are still unsatisfactory [5, 8]. In the past several years, a new procedure named renal denervation had been shown to be safe and effective in controlling hypertension, supporting that the sympathetic nervous system (SNS) activity is critical in regulating blood pressure [6, 7]. Recently, we have proposed a novel hypothesis that cervical spondylotic myelopathy may be an important etiology of essential hypertension which has been termed as cervicogenic hypertension [13]. In our previous study, we found that the high blood pressure in 12 out of 30 (40 %) hypertensive patients was reduced to normal levels following the cervical decompressive surgery, which resulted in a termination of the antihypertensive medications. Furthermore, the high and unstable blood pressure in the other 15 patients became stabilized and was well controlled by antihypertensive drugs after cervical decompressive surgery [13]. Recently, Peng et al. reported two cases of cervical spondylosis with hypertension [15]. After anterior cervical discectomy and fusion, the blood pressure of both the two patients remain normal without hypertensive medications [15]. Also, we speculate that renal denervation only destructs the network of SNS, and that the diseased cervical SNS has a greater impact on initiating and increasing SNS activity, as it may be closer to central nervous system than the normal renal SNS.

Through long–term clinical observations, we have found that high blood pressure or fluctuated blood pressure among many patients decreased substantially or became stable post-operatively. Therefore, we infer that cervical decompressive surgery may be a novel alternative in treating hypertension and will effectively reduce blood pressure in patients with cervicogenic hypertension. Therefore, a substantial number of patients with both hypertension and cervical spondylosis may benefit from this approach as the decrease in the quantity and/or dose of antihypertensive drugs would subsequently lead to fewer side effects from them. Regarding the rationale for the reduction on blood pressure, we speculate that the compression or irritation of the dura mater and the posterior longitudinal ligament of the cervical spine may increase the sympathetic nervous activation. This discharge may pass through the ganglia and the sympathetic trunk to the postganglia fibers arriving at the target organ, such as the vertebral artery and the blood vessels and subsequently induce hypertension. Decompressive cervical surgery, either anterior cervical discectomy and fusion or posterior laminoplasty, may withdraw any compression of the dura and the posterior longitudinal ligament and thus the sympathetic irritation will be relieved leading to a decrease of blood pressure.

Our study has a number of limitations. This is not a randomized controlled clinical trial, factors such as placebo and Hawthorne effects cannot be ruled out entirely. However, we tried to measure blood pressure two times both before and after the surgery, these multiple measurements would help to better understand if the change in blood pressure is attributed to the surgery or purely to the regression to the mean. Although the randomized controlled trial is the gold standard study design, it is unethical to conduct such a trial considering the inappropriateness of treating hypertension with a cervical decompressive surgery. In our opinion, the study still gains important scientific information. Once our hypothesis is approved, it will provide an innovative mechanism of hypertension development, which may lead to the development of new methods of hypertension treatment and prevention.
